# Identification and characterization of a novel *Enterococcus* bacteriophage with potential to ameliorate murine colitis

**DOI:** 10.1038/s41598-021-99602-4

**Published:** 2021-10-12

**Authors:** Junko Nishio, Hideo Negishi, Mika Yasui-Kato, Shoji Miki, Kazuhiko Miyanaga, Kotaro Aoki, Takuma Mizusawa, Masami Ueno, Akira Ainai, Masafumi Muratani, Sho Hangai, Hideyuki Yanai, Hideki Hasegawa, Yoshikazu Ishii, Yasunori Tanji, Tadatsugu Taniguchi

**Affiliations:** 1 Department of Molecular Immunology, Institute of Industrial Science, The University of Tokyo, 4-6-1 Komaba, Meguro-ku, Tokyo 153-8505 Japan; 2 Department of Inflammology, Research Center for Advanced Science and Technology, The University of Tokyo, 4-6-1 Komaba, Meguro-ku, Tokyo 153-8904 Japan; 3 Department of Immunopathology and Immunoregulation, Toho University School of Medicine, 5-21-16 Omori-nishi, Ota-ku, Tokyo 143-8541 Japan; 4 School of Life Science and Technology, Tokyo Institute of Technology, 4259 J3-8 Nagatsuta-cho, Midori-ku, Yokohama, Kanagawa 226-8501 Japan; 5 Department of Microbiology and Infectious Diseases, Toho University School of Medicine, 5-21-16 Omori-nishi, Ota-ku, Tokyo 143-8541 Japan; 6 Central Institute for Experimental Animals, 3-25-12 Tonomachi, Kawasaki-ku, Kawasaki, Kanagawa 210-0821 Japan; 7 Department of Pathology, National Institute of Infection Diseases, 1-23-1 Toyama, Shinjuku-ku, Tokyo 162-8640 Japan; 8 Department of Genome Biology, Faculty of Medicine, University of Tsukuba, 1-1-1 Tennodai, Tsukuba, Ibaraki 305-8575 Japan; 9 Present Address: Division of Vaccine Science, The Institute of Medical Science, The University of Tokyo, 4-6-1 Shirokanedai, Minato-ku, Tokyo 108-8639 Japan

**Keywords:** Mucosal immunology, Phage biology, Gastrointestinal diseases, Dysbiosis, Inflammatory bowel disease

## Abstract

Increase of the enteric bacteriophages (phage), components of the enteric virome, has been associated with the development of inflammatory bowel diseases. However, little is known about how a given phage contributes to the regulation of intestinal inflammation. In this study, we isolated a new phage associated with *Enterococcus gallinarum*, named *phiEG37k*, the level of which was increased in C57BL/6 mice with colitis development. We found that, irrespective of the state of inflammation, over 95% of the *E. gallinarum* population in the mice contained *phiEG37k* prophage within their genome and the *phiEG37k* titers were proportional to that of *E. gallinarum* in the gut. To explore whether *phiEG37k* impacts intestinal homeostasis and/or inflammation, we generated mice colonized either with *E. gallinarum* with or without the prophage *phiEG37k*. We found that the mice colonized with the bacteria with *phiEG37k* produced more Mucin 2 (MUC2) that serves to protect the intestinal epithelium, as compared to those colonized with the phage-free bacteria. Consistently, the former mice were less sensitive to experimental colitis than the latter mice. These results suggest that the newly isolated phage has the potential to protect the host by strengthening mucosal integrity. Our study may have clinical implication in further understanding of how bacteriophages contribute to the gut homeostasis and pathogenesis.

## Introduction

The gut microbiota is composed of hundreds of trillions of bacteria, fungi and viruses. In addition to supporting nutrient metabolism, recent studies have shown that commensal bacteria and, to some extent, fungi contribute to intestinal tissue homeostasis and maturation of the immune system^1,2^. On the other hand, roles of commensal *viruses* remain largely elusive. Most gut commensal viruses are bacteriophages (hereafter, phages), which infect bacteria. It has been speculated that the human gut is colonized with 10^9^–10^10^ phage particles per gram of feces, which is the largest group behind bacteria^3,4^. Therefore, it is likely that as yet unrecognized mechanisms of interaction operate between mammals and phages in the gut in health and diseases. In this context, numerous reports have pointed to the involvement of phages in intestinal pathogenesis^5–8^, however, little is known about the role of individual phage in either protective or harmful response.

Recent advances in high throughput metagenomic sequence technology have allowed us to more deeply investigate the complexity and richness of gut phage populations, the phageome^4,9,10^. In fact, a close link between an increase in gut phage richness and inflammatory bowel diseases (IBD) such as Crohn's disease and ulcerative colitis, which was accompanied by a decrease of the diversity of the bacterial populations has been demonstrated^8^. However, unlike the microbiome and mycobiome (fungi microbiome), the technology of analyzing the phageome is underdeveloped for several reasons. One of them is that many phages have two life cycles, a lytic cycle and lysogenic cycle. In contrast to the former in which host cell infection and lyses for viral dissemination is mediated by packaged, lytic virus-like particles (VLP), the latter cycle is defined by phage genome integration into target bacterial DNA or exosomal cytoplasmic plasmid in microbial host cells^11^. Although a recent increase in sequencing power has allowed for identification of many temperate phages, it is still difficult to understand the dynamics of changes in their life cycles, during which the phage exerts direct effects on host bacteria and possibly on the gut ecosystem. Additionally, databases for the phage sequences currently cover only around 20% of the sequences identified as phages^9^.

*Enterococcus* is a common member of intestinal microbiota and is present across a wide range of taxa, from insects to humans^12^. Though not inherently virulent, their properties give them a selective advantage in diverse environments. As a result, they out-compete other species when antibiotics are over-used or the host is immunocompromised^12^. Overgrowth of *Enterococcus* may potentially lead to their translocation into the host tissue, causing gut abscess, bacteremia and endocarditis. *Enterococcus* species are more enriched in the gut of IBD patients and treatments with biologics were shown to reduce their abundance^13,14^. Several recent papers have shown that *Enterococcus*-related phages also increased during colitis in humans as well as mice^5,8^. However, the roles of these *Enterococcus*-related phages in the disease are poorly understood.

In this study, we isolated phages from mouse intestinal content. Using autologous fecal *Enterococcal* bacteria, we screened for VLPs by phage plaque assay. A novel phage, termed *phiEG37k* was then identified by its association with *Enterococcus gallinarum*, whose frequency was found to be increased in mouse experimental colitis models. We then characterized the *phiEG37k* infectivity against the host and identified the life cycle within the intestinal ecosystem. Furthermore, by inducing colitis in the mice colonized with *phiEG37k* together with its host bacteria, we demonstrate the enhancement of colonic MUC2 by the phage and its ameliorating effect on colitis. We discuss these findings in the context of how bacteriophages contribute to gut homeostasis and pathogenesis.

## Results

### Screening of intestinal phage by plaque assay

**Figure 1:**
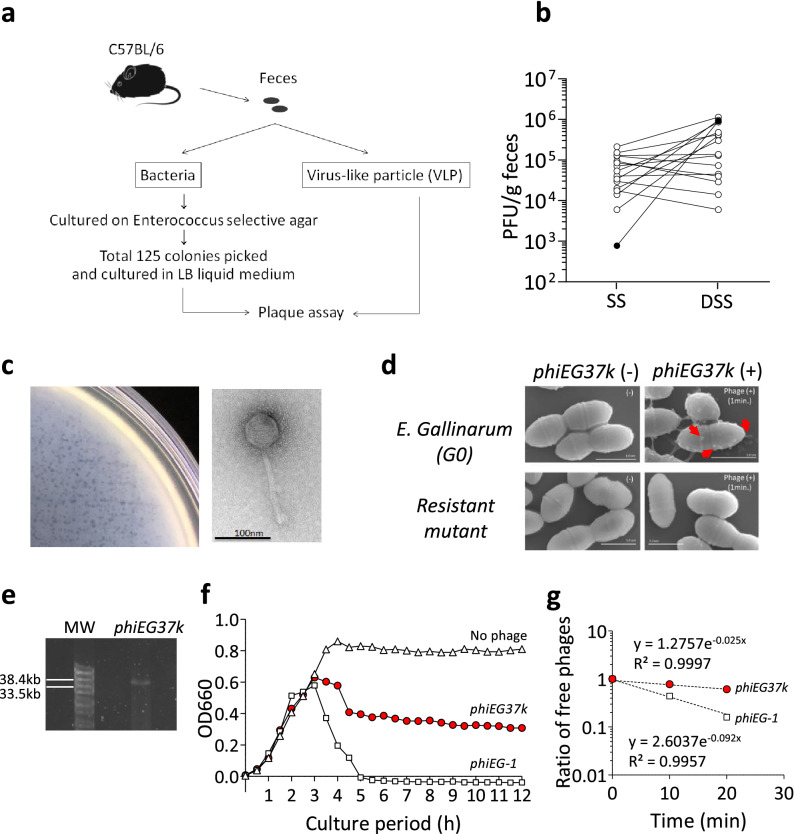
Identification of novel *Enterococcus* phages associated with colitis. Colitis-associated Enterococcus phages were identified by screening feces from C57BL/6 mice using a plaque assay. (**a**) Schema of screening for gut *Enterococcus* phages from C57BL/6 mice in steady state. (**b**) Plaque-forming unit (PFU) of phages associated with 16 individual Enterococcal bacterial clones was measured in steady state (SS) and during dextran sulfate (DSS)-induced colitis. The feces during colitis was taken on day 10, which is 3 days after the replacement of DSS with normal drinking water. The PFUs of the phage associated with *Enterococcus* strain G0 (*phiEG37k*) are indicated with black circles. (**c**) Plaques formed by *phiEG37k* phage (left panel) and the transmission electron microscopic image of the phage (right panel). (**d**) Scanning electron microscopic images of interaction of *phiEG37k* with host *E. gallinarum* or a resistant strain derived of the original strain. *phiEG37k* phages attached to the *E. gallinarum* are indicated by red arrows. Scale bars indicate 1 µm. (**e**) Electrophoresis of *phiEG37k* genome DNA on 1% agarose gel. MW; molecular weight marker (**f**) Lysis curves of *E. gallinarum* co-cultured with *phiEG37k* or *phiEG-1*, or without phage. The OD660 was measured on indicated time points. (**g**) Adsorption assay of *phiEG37k* and *phi*EG-1.

To isolate intestinal resident phages, we conducted a plaque assay using VLP fraction from the feces of C57BL/6 mice with individually isolated bacterial clones from the same mice (Fig. 1a). Since *Enterococcus* phage levels are notably increased in mouse colitis and human IBD^5,8^, we aimed at isolating *Enterococcus*-associated phages, which may affect the disease development. Sixteen of 125 fecal *Enterococcus* bacterial clones from six mice consequently were sensitive to infection by fecal VLP. Eleven of the 16 bacterial colonies showed an increase in the numbers of their related phages in the feces during dextran sulfate sodium (DSS)-induced colitis (Fig. 1b). Most notably, a phage related with one of the *Enterococcus* bacterial strains (*G0*) displayed a thousand-fold increase, with the highest increasing rate during colitis (Fig. 1b and Supplementary Fig. S1 online). After confirming the reproducibility of the increase of the *G0* strain-related phage during the DSS-induced colitis, we decided to further investigate the role of this phage in this disease model.

### Characterization of the isolated phage and its host

We then isolated the *G0*-associated phage which shows 1000-fold increase in colitis (Fig. 1b and Supplementary Fig. S1 online) and subjected it to electron microscopy analysis. The transmission electron microscope (TEM) analysis revealed that it is a long-tailed phage, which morphologically belongs to the family *Siphoviridae* in the order *Caudovirales* and is usually a dsDNA phage (Fig. 1c). The scanning electron microscope (SEM) analysis also revealed that the phage attaches to the surface of host *Enterococcus* bacteria, whereas it failed to attach to a phage-resistant mutant generated from the original host strain *G0* (Fig. 1d). The genome size of the phage was found to be about 37 k bp by Pippen pulse electrophoresis (Fig. 1e and Supplementary Fig. S2 online).

The whole genome sequence analysis revealed that this is a novel phage consisting 37,025 bp (Supplementary Table S1 online). Further, we identified that the host bacterium is *E. gallinarum*. We then termed this *E. gallinarum*-associated phage *phiEG37k*. The RAST analysis of predicted amino acid sequences based on the genome sequence revealed that *phiEG37k* possesses phage structural protein genes including major capsid protein, tail protein and tail length tape-measure protein (Supplementary Table S1 online). In addition, we detected the presence of the gene encoding phage lysin N-acetylmuramoyl-L-alanine amidase, an enzyme that hydrolyzes the phage-bacterial cell wall upon release of its progeny. Notably, *phiEG37k* also possesses an integrase gene, indicating that this phage is a temperate phage (Supplementary Table S1 online). Consistently, we found a gene encoding a RinA family phage transcriptional regulator required for the expression of the morphogenetic and lysis modules of the phage^15^ (Supplementary Table S1 online).

To further characterize *phiEG37k*, we next evaluated the lytic and adsorption activities of the phage. The lysis curve showed that *phiEG37k* only partially lysed the host bacteria and reached equilibrium between host growth and lysis in liquid culture within 4 h of coincubation of the *phiEG37k* and *E. gallinarum* (Fig. 1f). In contrast, *phiEG-1*, another *E. gallinarum*-related phage isolated from sewage, has strong lytic ability and completely killed the host. The adsorption ability of *phiEG37k* on the host is about 25% lower than that of *phiEG-1* (Fig. 1g). These results suggest that the *phiEG37k* phage and its host bacteria are capable of stably coexisting with each other in the gut ecosystem.

### Abundance of *phiEG37k* in colitis in mice

**Figure 2:**
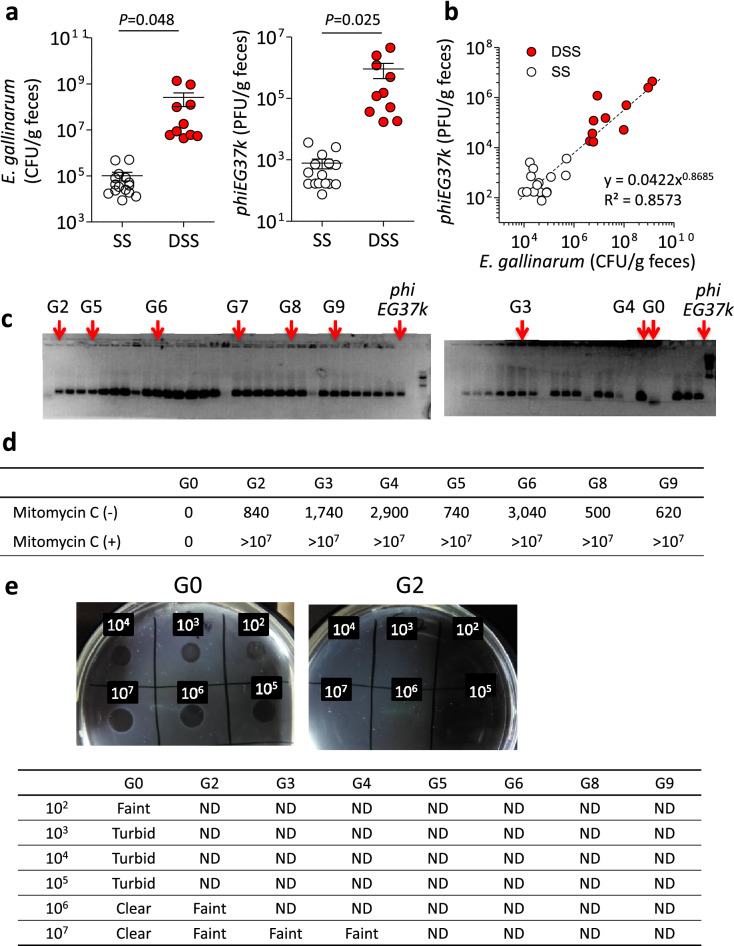
Most *phiEG37k* phages exist in lysogenic cycle in the colonic ecosystem in SPF C57BL/6 mice. The characterization of the interaction of *phiEG37k* phage and host *E. gallinarum* in the gut ecosystem was investigated*.* (**a**) Titers of *E. gallinarum* (left panel) and *phiEG37k* (right panel) in feces in steady state (SS) or DSS colitis (DSS). (**b**) Correlation curve of *E. gallinarum* titer *versus* *phiEG37k* titer in feces. n = 10. (**c**) PCR analyses of picked colonies of *E. gallinarum* isolated from feces of C57BL/6 mice in steady state by *phiEG37k*-specific primer. (**d**) Titer of *phiEG37k* phage particles in supernatant of individual cultures of different *E. gallinarum* colonies in **c** in the absence or presence of mitomycin C. (**e**) Spot assays of *phiEG37k* against individual clones in (**d**). ND; not detected. Two-tailed unpaired t-test *P* values are presented.

We next examined the potential role of *phiEG37k* in intestinal pathogenesis. In parallel with an increase in the number of *phiEG37k* during DSS-induced colitis in mice, the numbers of *E. gallinarm* were increased with the development of colitis (Fig. 2a). The correlation coefficient 0.8573 showed strong positive linear relationships between the phage and *E. gallinarm* titers both in the steady state and during DSS colitis development (Fig. 2b). The phage to microbe ratio (PMR) is about 1:1,000 which is considerably low (Fig. 2b). In general, phages prefer a lysogenic life cycle over a lytic cycle so as to take advantage of the bacterial proliferation in order to effectively propagate themselves in many ecosystems^16^. We then attempted to isolate 50 different colonies of *E. gallinarum* from the feces of C57BL/6 mice in steady state as well as after the onset of DSS colitis and subjected them to PCR analysis for the *phiEG37k* genome. Consistent with the above view, the *phiEG37k* genome was amplified in 44 of 50 colonies in steady state and in all 16 colonies of *E. gallinarum* after DSS colitis (Fig. 2c and Supplementary Fig. S3 online).

We next compared draft-whole genome sequences between randomly chosen *phiEG37k*-containing *E. gallinarum* strains, *G2*, *G3*, *G4* and *G6* (see Fig. 2c), with the original strain *G0* (*phiEG37k*-free) and confirmed that the phage sequence was integrated at the same site in the genome of all four strains (Supplementary Fig. S4 online). We performed phylogenetic analysis of *G0* and the four lysogenic strains (*G2*, *G3*, *G4* and *G6*) with a type strain *E. gallinarum* NCTC12359 (accession no. GCA_900447935.1) based on single nucleotide polymorphisms (SNPs) in the core-genome that is defined as DNA regions of genome shared by these compared strains. It revealed that *G0* and all the lysogenic strains formed a cluster with sharing 81.6% of the core-genome of NCTC 12359 (2,910,014/3,564,437 bp, Supplementary Fig. S5a online). As shown by the radial phylogenetic tree (Supplementary Fig. S5b online), the core-genome of the four lysogenic strains was 100.0% (3,563,364/3,564,437 bp) shared with *G0*, with the number of SNPs ranging from 30 to 69 between neighboring strains without shared SNPs, indicating that these strains divided from a common ancestor, which have not been isolated in this study^17^ (Supplementary Fig. S5b, c online).

As expected, all of the above and some additional lysogenic *E. gallinarum* strains (*G2*, *G3*, *G4*, *G5*, *G6*, *G8* and *G9*) were able to produce the lytic phage in liquid culture, the levels of which are dramatically augmented by mitomycin C treatment which induces a lytic phage release (Fig. 2d). Given that the PMR remained low and most fecal *E. gallinarum* bear lysogenic *phiEG37k*, most *phiEG37k* phage appear to exist largely in the lysogenic cycle in the mouse gut ecosystem. Of note, all of the *E. gallinarum* carrying the lysogenic phage displayed resistance to *phiEG37k* infection (Fig. 2e), supporting the notion that the ratio of lytic *phiEG37k* to *E. gallinarum* is relatively stable in the gut.

### Evaluation of the impact of *phiEG37k* colonization on intestinal homeostasis

**Figure 3:**
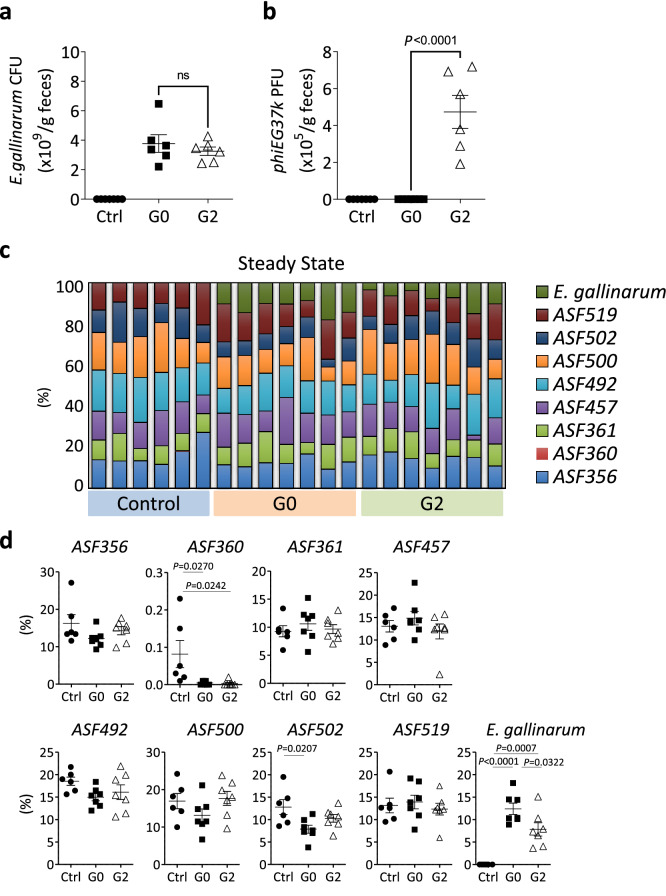
Stable colonization of *E. gallinarum* bearing lysogenic *phiEG37k* phage produces residual phage particles in the colon. C57BL/6 mice that have Altered Shaedler Flora (ASF-mice) were gavaged *E. gallinarum* either free from (*G0* strain) or bearing lysogenic *phiEG37k* (*G2* strain). (**a** and **b**) Titers of *E. gallinarum* (**a**) and *phiEG37k* (**b**) in feces from the ASF mice with no *Enterococcus* colonization (Ctrl), *G0*-colonization or *G2*-colonization at 4 weeks post-gavage. (**c**) Components of colonic microbiota in mice of the three groups described in (**a**). (**d**) Abundancy of individual bacteria consisting of ASF or *E. gallinarum* in the colon from mice described in (**a**) was displayed. One way analysis of variance with Tukey’s multiple comparison was performed for ad-hoc analysis. *ns*; not significant. n = 6–7.

To explore how gut colonization of *E. gallinarum* infected by *phiEG37k* affects the host’s intestinal pathogenesis, we first attempted to generate mice colonized with the host bacteria alone (*G0* strain) or mice colonized with the *G2* strain which is a lysogeny strain containing *phiEG37k* genome by oral gavage. First, C57BL/6 mice were purchased from one of the mouse suppliers whose mouse colonies are free from *phiEG37k* (Supplementary Fig. S6 online). However, the colonization of both strains (*G0* and *G2*) turned out unsuccessful; this is probably due to colonizing resistance caused by the resident microbiota^18^. Therefore, as an alternative approach, we chose mice colonized with Altered Shaedler Flora (ASF) consisting of eight known symbiotic commensal bacterial species but without *Enterococcus* bacteria (hereafter ASF mice)^19^. We then found that both the phage-free *E. gallinarum G0* and the *phiEG37k*-positive *E. gallinarum G2* were stably colonized for longer than 4 weeks after inoculation by oral gavage to these mice (Fig. 3a, b).

In further characterization of these ASF mice with *E. gallinarum*, we confirmed by whole genome sequencing analysis that *G0* and *G2* strains had only 60 single nucleotide polymorphisms on the core-genome (Supplementary Table S2 online). *Enterococcus* titers were equivalent in the feces between the two mouse strains (Fig. 3a). There was no significant alteration in abundance of the eight individual bacterial components of ASF between the mice colonized with *G0* and *G2* strains in steady state, although there was a slight decrease in that of *E. gallinarum* in the *G2*-coloinized mice (Fig. 3c, d). The components of ASF as well as *E. gallinarum* were not altered significantly during colitis between the mice colonized with *G0* and *G2* strains (Supplementary Fig. S7 online).

To explore how the immune cell phenotype is influenced by gut colonization with *E. gallinarum* with or without *phiEG37k* phage, i.e., *G2* and *G0* strain, flow cytometric analysis was performed for T cells and dendritic cells from lamina propria. There was no observed significant difference in cell frequencies or expression of activation markers between the mice colonized with *G0* and *G2* strains (Supplementary Fig. S8 online). Interestingly, however, comparative analysis for gene expression profiles of whole colon tissues between these mice revealed that thirty-four genes were significantly changed in the mice colonized with *G2* strains, *i.e. phiEG37k*-colonized mice (Supplementary Fig. S9 and Table S3 online). Interestingly, mRNAs for a series of mucin genes including *Muc2*, *Muc3*, *Muc3a* and *Muc4* were significantly elevated in the colons from the *phiEG37k*-colonized mice as compared to those from uncolonized mice (Supplementary Fig. S9 and Table S3 online).

**Figure 4:**
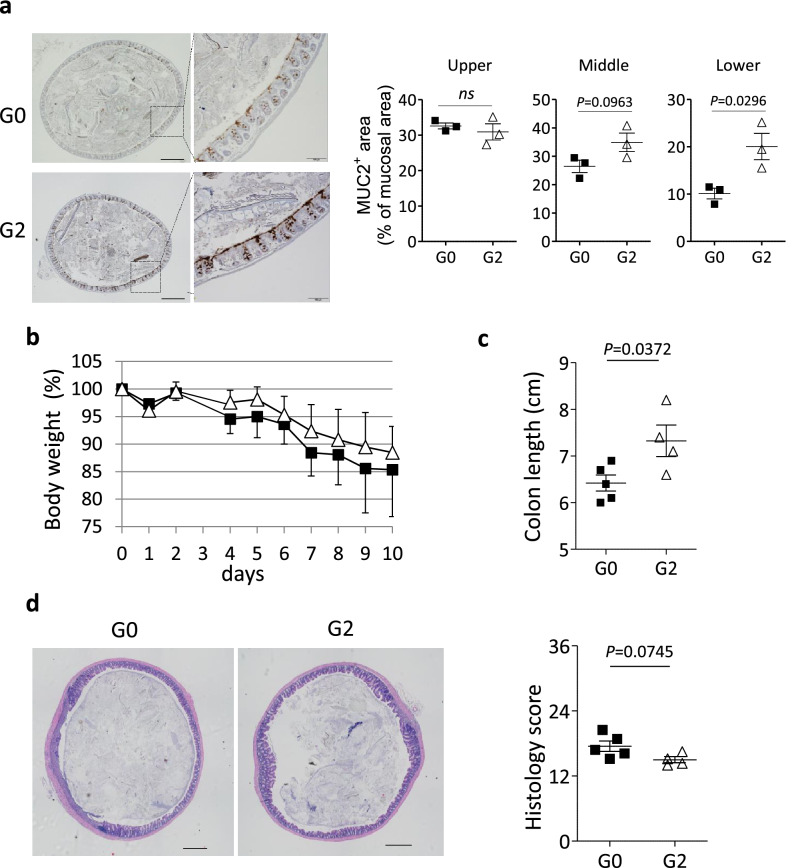
*Enterococcus* phage colonization potentially enforces colonic barrier function. The role of *phiEG37k* in gut homeostasis and inflammation was explored by analyzing the ASF-mice colonized with *G0* or *G2*. (**a**) Immunohistochemical analysis for MUC2 protein in the colonic tissue from ASF-mice colonized with *G0* or *G2* (n = 3 per each group). Representative images of lower colons from mice colonized with *G0* or *G2* (left panel). The percentage of MUC2-stained area in mucosal area (right panels) was quantitated by ImageJ software. Scale bars indicate 500 µm in left panels and 100 µm in right panels. (**b**–**d**) 2.5% of DSS was administered for 7 days to *G0*- or *G2*-colonized mice for colitis induction (n = 4–7 per each group). Two independent experiments were performed. (**b**) Body weight change during DSS colitis (n = 5 for *G0*, n = 4 for *G2*). (**c**) Colon length on day 9 post-administration with DSS is shown. (**d**) Representative HE stained colonic tissue (left panels) and histology scores of colitis (right panel) from mice described in (**b**). Scale bars indicate 500 µm. Two-tailed unpaired t-test *P* values are presented. *ns*; not significant. n = 3 in (**a**). n = 4–5 in (**c**) and (**d**).

Mucin proteins are major components of mucus, which physically covers the surface of intestinal epithelial cells to protect from invasion of commensal or pathogenic bacteria^20–22^. In fact, immunohistochemical analysis revealed an elevation of MUC2 production in the colonic epithelial cells from *phiEG37k* -colonized mice compared with those from uncolonized mice (Fig. 4a). Thus, these results suggest that the *phiEG37k* colonization potentially enhances the barrier of intestinal mucosa through higher levels of MUC2 production.

As an approach to explore whether *G2* or *phiEG37k* directly enhances mucin genes, we incubated the human colonic epithelial cell line Caco-2 with *E. gallinarum G0*, lysogenized strain *G2* or *phiEG37k.* However, *MUC3* mRNA expression was not significantly elevated in Caco-2 cells co-cultured with *G2* or *phiEG37k* compared to that with *G0* (Supplementary Fig. S10 online). Caco-2 cells lack the *MUC2 gene* and the *MUC4* mRNA level was too low to quantify expression levels. Although the result is seemingly inconsistent with that in *G2-*colonized mice, we infer that the environment of gut lumen and mucin in vivo cannot be reproduced in vitro*.* In the gut mucosa, more intensive bacterial lysis may occur by phage infection or by a life cycle switch from lysogenic to lytic, which leads to exposure of bacterial antigens and/or pathogen-associated molecular pattern molecules to the gut epithelial and dendritic cells. Alternatively, it may be ascribed to the difference between cell lines and primary cells, or difference between cells from human and mice in terms of the cell’s responsiveness to the phage. Finally, we cannot exclude the possibility that the mRNA induction in vivo by *phiEG37k* may be indirect in that yet unknown factors may be mediating the mRNA induction by the phage. This is obviously in interesting issue to be addressed in the future study.

### Effect of the *phiEG37k*-containing bacteria in colitis

We next examined the ASF mice with or without the phage colonization in DSS-induced colitis model. Both mice colonized with *G0* and the phage-containing *G2* strains were healthy and had no signs of colitis at least 4 weeks post-colonization. These mice were then treated with 2.5% of DSS. The symptoms of DSS-induced colitis are characterized by body weight loss and bloody diarrhea. Although no significant difference in body weight changes was detected between the colonized mice of the *G0* strain and *G2* strain (Fig. 4b), we observed significant differences in colon length after 9 days post-induction of colitis. The *G2* strain-colonized mice displayed longer colons than uncolonized mice, indicating that inflammatory changes in the colon tissue, such as infiltration or edema, are ameliorated by the *G2* strain colonization (Fig. 4c). Further, histological analysis revealed a broader area of colonic mucosa was inflamed in *G0* strain-colonized mice than in *G2* strain-colonized mice (Fig. 4d, left), Histology scores were substantially lower for the *G2* strain-colonized mice compared to those of the *G0* strain-colonized mice (Fig. 4d, right). Taken together, *phiEG37k*-containing *G2* strain colonization displayed a mild ameliorating effect on DSS-induced colitis. This effect may be attributed to the enhanced production of MUC2 by colonization with *G2*.

## Discussion

In this study, we isolated and characterized a novel gut-resident phage *phiEG37k* and its host *E. gallinarum* by screening based on lytic activity of the VLP fraction of feces from C57BL/6 mice. We found that the lytic *phiEG37k* resides as phage particles in the gut across C57BL/6 mice. Both gut *E. gallinarum* and *phiEG37k* phage were increased during colitis, and their titers had a strong correlation with very low PMR across the mice in steady state as well as during colitis. We also found that a majority of intestinal *E. gallinarum* in C57BL/6 mice bore lysogenic *phiEG37k* in their host genome, which also displayed resistance to *phiEG37k* infection. These results suggest that a small fraction of host lysogens release *phiEG37k* phage particles due to the change of their life cycle from lysogenic to lytic state.

We successfully generated mice colonized with the host *E. gallinarum* with the lysogenic *phiEG37k* phage (*G2* strain) and without the prophage (*G0* strain). The *G2*-colonized mice stably displayed the *phiEG37k* lytic phage in the gut. Furthermore, we found that these mice are more resistant to experimental colitis, compared to the *G0* -colonized mice, suggesting the ameliorating effect of *phiEG37k* on colitis. The enhancement of MUC2 production in the colonic epithelial cells from *phiEG37k*-colonized mice (Fig. 4a) may account for the ameliorating effect of the *phiEG37k* phage in this colitis model.

Our study elucidated a mechanism for increased phage particles during colitis that occurred as a result of an increase in the number of host lysogens releasing lytic phage in the gut. Few studies have focused on intestinal resident phage from the point of view of the different life cycles of the phage and its interaction with the host bacteria in the gut ecosystem. Very recent progress of metagenomic analysis has allowed us to characterize host-phage relationships beyond information about diversity and abundance of bacteriophage populations^6,23,24^. However, we have not been able to trace their life cycle changes or interactions with host bacteria. In this regard, our study could provide new findings about the biology of gut phage.

The lower value of PMR indicates low production of phage particles and suggests that the phage is in the state of lysogeny^25^. The PMR of *phiEG37k* to *E. gallinarum* ≈ 0.01 is much lower than that observed in the sea and soil where lytic phage is dominant, 2.6 to 160^24^. This suggests that most *phiEG37k* phage resides in the lysogenic cycle and a very small fraction of the *phiEG37k* switches from the lysogenic to the lytic cycle resulting in the release of phage particles from host cells. It is also speculated that host *E. gallinarum* is comprised of a mixed population of dominant lysogens bearing *φEG37k* prophage with very few prophage-free cells in the mouse gut^25^.

Host bacteria that were once infected with certain phages, become resistant to the phage through multiple mechanisms^26^. A majority of *E. gallinarum* colonies in the feces from C57BL/6 mice were observed to bear prophage *phiEG37k*, indicating that they had been previously infected with *phiEG37k* (Fig. 2c). It has been hypothesized that host bacteria killed by gut residual phage contributes to the maintenance of microbiota composition by inhibiting excessive expansion of certain host bacteria, however, this is not the case for certain bacteria that are lysogens and resistant to the infection by the related phage as *E. gallinarum* lysogen is resistant to *phiEG37k* (Fig. 2e).

It is interesting that mice colonized by *phiEG37k* phages are more resistant to DSS-induced colitis (Fig. 4b). This result suggested an increase in lytic *phiEG37k* phage particles was not disease initiating, but the consequence of gut inflammation under which *E. gallinarum* robustly expanded. Multiple different phages increase in the gut during gut inflammation in mice as well as in humans^5,8^. Thus, we speculate the real impact of the phageome on colitis could be a consequence of the accumulation of a variety of effects by multiple phages. Some phages could have colitogenic effects in colitis, while others have a role as exemplified in this study with *phi*EG37k, which is involved in the enhanced production of MUC2. In this regard, adherent invasive *Escherichia coli*–related phages, which directly stimulate immune cells resulting in exacerbation of colitis, might be an example as shown in a recent paper^7^.

Another interesting question not answered by our study is how *phiEG37k* functions in the production of MUC2. Since the expression of MUC2 proteins and mRNA are enhanced in the *phiEG37k*-colonized colon, epithelium cells might have continuously received signaling from altered mucus environments generated by *phiEG37k* colonization. Another possibility is that the *phiEG37k* phage particles engulfed by dendritic cells in the lamina propria may have indirectly stimulated epithelial cells to enhance mucin production, as shown in the recent study^7^. It is also possible that the *phiEG37k* phage particles might localize on the surface of the mucus layer, as previously reported^27^. In this report, a “bacteriophage adherence to mucus” model was proposed in that phages have an immunoglobulin (Ig)-like structure in the tail and loosely bind to the surface of mucus through this structure which limits the number of intestinal bacteria able to penetrate the mucus layer. Thus, following this model, the lytic *phiEG37k* phage might be concentrated on the mucus surface and lysed host bacterial cells, leading to the alteration of mucus components of the cell fragments, including bacterial cell walls and DNA. Further study is obviously required to clarify this point.

We here propose the scenario that *phiEG37k* prophage-bearing *G2* enforces mucin production and alleviates murine colitis. However, since *G0* and *G2* are genetically 100.0% identical except for the insertion of *phi37kEG* genome and have nonsynonymous SNPs between them (Supplementary Table S2 online), we cannot rigorously exclude the possibility that the difference in *E. gallinarum* strain-specific characteristics might result in different sensitivity to colitis. Further study will be required to impeccably clarify this issue.

In summary, we identified an *E. gallinarum*-associated novel temperate phage, *phiEG37k*, which is colonized in the mouse gut and increases with experimental colitis, and adduced evidence for its potential to protect the host from colitis. Exact mechanism(s) of how this phage actually contributes to the pathogenesis needs further investigation. In view of a close link between phage abundance and IBD^5,8^, one may speculate from our study that there may be distinct phage populations in IBD that affect either for disease exacerbation or suppression. If so, one could isolate protective phages and host bacteria for the disease amelioration and/or suppression. Such an effort may pave a way for a new rationale for phage therapy of the disease.

## Materials and methods

### Mice

Specific pathogen free C57BL/6 mice (n = 41) were purchased from Clea Japan, Inc or Charles River Laboratories Japan (n = 29). The screening of gut phage was conducted using C57BL/6 mice from Clea Japan. The feces for detection of *phiEG37k* phage was obtained within a week after the mice had shipped from the vendors. Germ-free C57BL/6 mice purchased from Clea Japan were colonized with Alter Schaedler flora (ASF mice) and maintained in vinyl-isolators at Central Institute of Experimental Animal (CIEA, n = 73). All animal experimental protocols under SPF condition were approved by the Institutional Animal Care and Use Committees of the University of Tokyo (Approval numbers:27-8 and RAC19003). Experiments using ASF-colonized mice were approved by the Institutional Animal Care and Use Committees of CIEA (Approval number: 19062A). All animal care and experiments were performed in accordance with guidelines of the University of Tokyo and those of CIEA. The experiments involving live animals were done by following the recommendation in the ARRIVE guidelines.

### Dextran sodium sulfate (DSS)-induced colitis

For colitis induction in SPF C57BL/6J mice, the mice were administered with 2.0% DSS (MP Biomedicals) in drinking water ad libitum for 7 days. Regarding the screening experiments, feces was taken 3 days after the administration of DSS was stopped. For ASF mice colonized with *E. gallinarum*, the mice were administered with 2.5% DSS in drinking water for 7–9 days 4 weeks after gavage with the bacteria. For body weight (BW) change studies, percent BW relative to that at day 0 was determined.

### Preparation of* Enterococcus* host strains and phage source

We conducted screening for feces from 16 mice. One to two feces taken from individual C57BL/6 mice were pooled and suspended in SM buffer (100 mM NaCl/8 mM MgSO_4_/50 mM Tris–HCl/0.01% gelatin, pH7.5) at 0.025 mg/ml or 0.05 mg/ml. To obtain *Enterococcus* bacterial colonies, 100 µl was inoculated on BBL *Enterococcus* agar (BD) plate and cultured at 37 °C overnight. The colonies were picked next day and transferred in 100 µl of Luria–Bertani (LB) medium for culture overnight. On the third day, glycerol stocks of individual *Enterococcus* bacteria were generated and stored at − 80 °C. To obtain VLP, the supernatant of the remaining fecal suspension was collected by centrifuging at 8000 × *g* for 5 min and added with 10 µl of chloroform on the first day. The VLP suspension from individual mice was pooled and kept at 4 °C until used for assays. The overnight culture of individual *Enterococcus* bacteria from the glycerol stock and the VLP were submitted to plaque assays. To prepare VLP suspension from mice with colitis, colonic content obtained by cutting open the colon was suspended in SM buffer. To confirm the alteration in abundance of fecal VLP in colitis, the sixteen *Enterococcus* bacterial clones that were sensitive to fecal VLP infection at the first screening plaque assay were subjected to another set of plaque assays with VLP suspension obtained from another set of 10 mice in steady state and with DSS colitis.

### Plaque assays

For the screening, fifty or 100 µl of VLP was mixed with 100 µl of overnight culture from individual glycerol stocks of *Enterococcus* bacteria and then added in 3 ml of 45 °C-warmed LB soft agar (0.5% agar) and vortexed. The mixture was transferred to a petri dish and cultured at 37 °C. The number of plaques was counted the next day. For evaluation of titer of fecal *phiEG37k*-colonization in ASF mice, a piece of feces (0.01 to 0.035 g) was suspended in 1 ml of SM buffer and 10 µl of fecal VLP was mixed with 100 µl of overnight culture from glycerol *Enterococcus gallinarum* (*G0* strain) for the plaque assay as described.

### Isolation and purification of* phiEG37k*

The *phiEG37k* was isolated from a single plaque formed at the plaque assay described above. The plaque together with host bacteria in the soft agar was inoculated in 2 ml of LB media buffered with SM buffer (LB/SM media) overnight. The small culture was transferred to the large volume culture in LB/SM media containing freshly cultured *E. gallinarum* suspension and cultured overnight. The next day the cell suspension was centrifuged at 8000 × *g* for 5 min and one drop of chloroform was added to the collected supernatant. Gradient precipitation by CsCl (1.46, 1.55 and 1.63 g/ml) was conducted to isolate a large number of phage particles. To exchange the buffer, dialysis was performed with SM buffer.

### Transmission electron microscope (TEM) analysis for the phage

Purified phages diluted in PBS were allowed to adhere to 300-mesh carbon-coated Cu grids (Veco grids; Nisshin EM, Tokyo) for 1 min at room temperature, and then negatively stained with 2% uranyl acetate solution. TEM analysis was performed using H-7650 (HITACHI, Tokyo, Japan).

### Scanning electron microscope (SEM) analysis

*Enterococcus gallinarum* were infected with 10 multiplicity of infection (MOI) of *phiEG37k* for the indicated time periods in LB media and fixed with 2% paraformaldehyde and 2.5% glutaraldehyde in PBS, followed by post fixation with 1% OsO_4_ in the same buffer. The fixed samples, which were placed on poly-L-lysine-coated coverslips, were washed three times with PBS, dehydrated in graded acetone solutions (50, 70, 90, 95, 99.5%), and then immersed in tert-butyl alcohol. After replacing the ethanol with tert-butyl alcohol, the samples were dried by using a freeze-drying device (VFD-21S; VACUUM DEVICE), and then coated with osmium by using a plasma coating device (Neoc; Meiwafosis). The samples were visualized with SEM (SU6600, Hitachi High-Technologies, Tokyo, Japan).

### Genome isolation and whole genome sequence for the* phiEG37k* and host *Enterococcal* bacteria

The genome of phages was extracted by PureLink Viral RNA/DNA Mini Kit (Invitrogen) by following the manufactural protocol. Bacterial genome was isolated by DNeasy Blood & Tissue (QIAGEN) by following the manufactural protocol. The whole genome sequence of purified phage and bacteria was analyzed by next generation sequencer (HiSeq, Illumina) with 150 bp paired-end sequencing at BGI genomics and Eurofins genomics, respectively. All sequenced data for the phage were assembled by SPAdes (http://cab.spbu.ru/software/spades/). The individual genes in the whole phage genome of *phiEG37k* (accession number; LC625742) were annotated by RAST server (http://rast.nmpdr.org). The host bacterium was identified as *E. gallinarum* by the Average Nucleotide Identity (ANI) analysis using draft whole-genome sequencing data.

### Core-genome single polymorphism analysis

To analyze the core-genome single-nucleotide polymorphism (SNP)-based genetic relationship among *E. gallinarum* strains, HiSeq sequencing data were aligned to the complete genome sequence of *E. gallinarum G0* draft genome using Burrows-Wheeler Aligner with the “MEM” option. SNP extraction was performed by SAMtools (version 1.1) mpileup and VarScan (version 2.3.7) mpileup2cns.

### Coculture of* phiEG37k* and* E. gallinarum*

Twenty µl of fresh confluent *G0* and 20 µl of 4 × 10^6^ *phiEG37k* particles were mixed to allow the phage adsorption on the host *G0*. To remove the unabsorbed phages, the mix was centrifuged at 8000 × *g* for 5 min and the supernatant was discarded. After washing twice, the host cell pellet resuspended in 4 ml LB was transferred to an L-shaped glass test tube and culture was started. For lytic curves, the optical density at wavelength of 660 nm (OD660) was monitored by TVS062CA a compact rocking incubator (Advantec, Tokyo, Japan) at 20-min intervals for a minimum of 9 h following phage addition. In some experiments, 0.5 µg/ml of mytomicin C was added to the bacterial culture to induce a curing of all lysogenic phage from the host cell.

### Adsorption assay

Adsorption efficiency of the *phiEG37k* on *E. gallinarum* was measured by titrating the presence of free phages in the supernatant under the low MOI condition (MOI = 0.01) for 20 min to evaluate the affinity of initial adsorption between a host cell and its phage.

### Determination of fecal titer of* phiEG37k* and *E. gallinarum*

One to two pieces taken from SPF C57BL/6 mice were weighed and suspended in 1 ml of SM buffer. One hundred µl or 10 µl of fecal suspension from C57BL/6 mice or ASF mice, respectively, was inoculated on an *Enterococcal* agar plate and cultured overnight. The next day, ten colonies were picked and subjected to PCR specific to *E. gallinarum* (see the following PCR methods)*.* The fecal titer of *E. gallinarum* was calculated based on the number of *E. gallinarum*-PCR positive colonies. The rest of the fecal suspension was centrifuged at 8000 × *g* for 5 min and the supernatant was used for the VLP solution. One hundred µl of VLP solution from SPF C57BL/6 mice or ten µl of VLP solution from ASF mice was submitted to a plaque assay with overnight cultured *E. gallinarum G0* strain. The fecal titer of *phiEG37k* was calculated based on the number of plaques by the assay.

### PCR for* E. gallinarum* and *phiEG37k*

To determine if individual bacterial colonies grown on the *Enterococcus* plate were *E. gallinarum*, colony-pick PCR was performed using Mighty Amp Polymerase III (Takara) with primer specific to *E. gallinarum*. The primer used was forward 5′-TTACTTGCTGATTTTGATTCG-3′; reverse 5′-TGAATTCTTCTTTGAAATCAG-3′. The PCR conditions were as follows; (1) an initial step of 2 min at 98 °C and (2) 32 cycles with a cycle consisting of 30 s at 94 °C, 1 min at 55 °C and 1 min at 72 °C. To determine whether the genome of *E. gallinarum* bear integrated *phiEG37k* genome, *E. gallinarm* colonies were picked and subjected to PCR with primer specific to *phiEG37k*: forward 5′-TGAGTAACATCAACATGGGCTTAG-3′; reverse 5′-ATATGCCACCTGCTTTCCTG-3′. The PCR conditions were as follows; (1) an initial step of 2 min at 98 °C and (2) 32 cycles with a cycle consisting of 30 s at 94 °C, 1 min at 60 °C and 20 s at 72 °C. The primer amplifies 130 bp size segment of genomic DNA at a site between bp 21,073 and 21,202, containing a 3′-end of BppU family phage baseplate upper protein and a 5′-end of hypothetical protein (Supplementary Fig. S11 online).

### Spot assay

Five microliters of phage suspension with indicated titers were dropped onto the LB soft agar plate mixed with 100 µl of overnight culture from glycerol stock of *E. gallinarum* (*G0* or *G2* strain). The phage resistance of individual strains of *E. gallinarum* was assessed by the clarity of plaques, which was scored as clear, turbid or faint.

### Colonization of the *phiEG37k* and* E. gallinarum*

5 × 10^8^ of either strain of *E. gallinarm* *G0* or *G2* was gavaged ASF-colonized C57BL/6 mice at 4 weeks of age. After 4 to 6 weeks, DSS-colitis was induced. To confirm the colonization of the phage and host bacterium, feces was collected before and at indicated time points after *E. gallinarum* administration and subjected to titer check for *phiEG37k* and *E. gallinarum*. The titer of *E. gallinarum* was determined by PCR as described above. The titer of free *phiEG37k* phage was determined by plaque assay with *E. gallinarum G0* strain.

### Microbiota composition of ASF mice

Bacterial DNA was extracted from ASF-mice, and *G0*- or *G2*-colonized ASF-mice using QIAamp DNA Stool Mini kit (Qiagen) by following manufactural protocol and submitted to quantitative PCR using primers for each bacterial component of ASF^19^ and for eubacterial 16S rRNA^28^. The PCR condition was described previously^19^.

### RNA-seq analysis

Whole colon was dissected from *G0*- and *G2*-colonized ASF mice and homogenized by polytron in TRIZOL (Thermo Fisher) after removing feces. Total RNA was extracted by following the manufactural protocol of TRIZOL. 500 ng of total RNA was used for rRNA depletion (New England Biolabs, E6310), followed by directional library synthesis by using Next® Ultra™ Directional RNA Library Prep Kit (NEB E7420). Sequencing was performed by NextSeq500 (Illumina). A paired-end run was conducted to obtain 2 × 36-base reads. FASTQ files were imported to CLC Genomics Workbench (CLC-GW, v10.1.1, Qiagen), mapped to mouse reference genome (mm10), and quantified for 49,585 genes. For statistical analysis, pairwise comparison was conducted with Empirical Analysis of DGE tool in CLC-GW. Differentially expressed genes were determined by a false discovery rate-adjusted *P*-value of less than 0.05.

### Isolation of lamina propria cells

The detailed procedure was previously described^29^ and is briefly summarized as follows. Whole colonic tissue was incubated in EDTA-containing buffer to remove IEC. The remaining lamina propria with muscles was cut into small pieces and digested with collagenase D (Roche), DNaseI (Roche) and dispase (Roche). After washing, cell suspension was subjected to gradient separation to obtain lamina propria cells in the layer between the upper and lower phase. The antibodies used were listed in Supplementary Table S4 online.

### Flow cytometric analysis

Cell surface staining was performed by standard procedures and reagents. Intracellular staining for cytokines and Foxp3 staining was performed using eBioscience™ Foxp3 / Transcription Factor Staining Buffer Set with manufacturer’s protocols (Invitrogen). Dead cells were eliminated in intracellular staining experiments by LIVE/DEAD® Fixable Dead Cell Stain Sampler Kit (Invitrogen). Analysis was performed using LSR Fortessa instrument (BD Biosciences) and FlowJo software (BD Biosciences). All antibodies used in the study are shown in the Supplemental Table S4 online.

### Culture of Caco-2 cell line

Caco-2, a human colonic epithelial cell line, was purchased from ATCC. When the cells plated in 24-well plates reached 80% confluence, *G0*, *G2* or *phiEG37k* was added in the following numbers; G0 or G3, 4 × 10^3^, 2 × 10^4^ and 1 × 10^5^ cells; *phiEG37k*, 4 × 10, 2 × 10^2^, 1 × 10^3^, 1 × 10^4^ and 1 × 10^5^ phage particles. After 4 h, the supernatant was discarded and 500 µl of RNA iso plus (TAKARA) was added to subject to RNA extraction.

### qRT-PCR

Total RNA from Caco-2 cells was extracted by following the manufactural protocol of RNAiso and reverse-transcribed with PrimeScript™ RT reagent Kit with gDNA Eraser (TAKARA). qRT-PCR was performed on Light Cycler 480 (Roche Bioscience) using the TB Green® *Premix Ex Taq*™ II (TAKARA) and values were normalized to the expression of *GAPDH* mRNA. Primer sequences are as follows: *GAPDH* forward 5′-CCTCCAAGGAGTAAGACCCC-3′; *GAPDH* reverse 5′-TGTGAGGAGGGGAGATTCAG-3′; *MUC3* forward 5′-CAGTCCCCCAGCCCTAAA-3′; *MUC3* reverse 5′-TATACCTTGCTAGGGACCAGGA-3′.

### Histological analysis

Isolated colons were fixed in 4% paraformaldehyde for 16 h at room temperature, embedded in paraffin and stained with hematoxylin and eosin (H&E). Immunohistochemical analysis of MUC2 was performed with rabbit polyclonal anti-MUC2 Ab (GENETEX, Inc) and HRP-conjugated secondary Ab to visualize signal using DAB substrate. The H&E or immunohistochemical sections were examined by automated fluorescence microscope BX63 (Olympus). Colitis histological scoring was performed by following a previous study^30^. Each section is assigned for scores based on the degree epithelial damage (None, 0; Loss of less than basal 1/3 of the crypt, 1; Loss of the entire crypt but intact surface epithelial cells, 2; Loss of both the entire crypt and the surface epithelial cells, 3) and inflammatory infiltration (None, 0; into the mucosa, 1; into submucosa, 2; into muscularis/serosa, 3). Each of the four scores multiplied by 1 if the change was focal, 2 if it was patchy, and 3 if it was diffuse, and the sum is considered as colitis histological scores per mouse which ranges 0 to 36.

### Statistical analysis

Statistical analyses were performed using Prism ver. 7.0 software (Graphpad Software, San Diego, CA, USA). An unpaired t-test was used for the two-group comparison. A one-way analysis of variance (ANOVA) was used for the three-group comparison and Tukey’s test was used for ad-hoc test. Two-tailed *p*-values less than 0.05 were considered significant. All data were expressed as the mean ± standard error (SEM).

## Supplementary Information

Supplementary Information 1.
