# Supplementary material for: Identification and characterization of a novel Enterococcus bacteriophage with potential to ameliorate murine colitis
Source: Sci Rep. 2021 Oct 12;11:20231. doi: 10.1038/s41598-021-99602-4 (PMC8511138; doi:10.1038/s41598-021-99602-4)
Supplement: Supplementary file 1 — Supplementary Information 1. [file 41598_2021_99602_MOESM1_ESM.pdf]

Supplemental information

Identification and characterization of a novel *Enterococcus* bacteriophage with potential to ameliorate murine colitis

Junko Nishio, Hideo Negishi, Mika Yasui-Kato, Shoji Miki, Kazuhiko Miyanaga, Kotaro Aoki, Takuma Mizusawa, Masami Ueno, Akira Ainai, Masafumi Muratani, Sho Hangai, Hideyuki Yanai, Hideki Hasegawa, Yoshikazu Ishii, Yasunori Tanji, Tadatsugu Taniguchi

CDS Position		Strand	Length (bp)	Function
360	34	-	327	prophage LambdaBa04, Gp54
577	350	-	228	hypothetical protein
1763	567	-	1197	Helix-turn-helix domain-containng protein
2400	1987	-	414	RinA family phage transcriptional regulator
2613	2401	-	213	hypothetical protein
2775	2614	-	162	hypothetical protein
2868	2776	-	93	hypothetical protein
3074	2865	-	210	hypothetical protein
3275	3075	-	201	hypothetical protein
3576	3265	-	312	hypothetical protein
3959	3573	-	387	Hypothetical protein, Lmo2313 homolog [Bacteriophage A118]
4004	4264	+	261	hypothetical protein
4477	4301	-	177	hypothetical protein
4821	4477	-	345	hypothetical protein
5140	4841	-	300	hypothetical protein
5324	5118	-	207	hypothetical protein
5587	5360	-	228	hypothetical protein
5935	5633	-	303	hypothetical protein
6190	5951	-	240	hypothetical protein
6542	6228	-	315	hypothetical protein
6772	6539	-	234	hypothetical protein
6912	6769	-	144	hypothetical protein
7244	6912	-	333	hypothetical protein
9884	7557	-	2328	DNA primase, phage associated
10408	9905	-	504	Single stranded DNA-binding protein
11772	10432	-	1341	Putative helicase
12452	11769	-	684	NTP-binding protein
12916	12449	-	468	hypothetical protein
13153	12917	-	237	hypothetical protein
13978	13214	-	765	hypothetical protein
14239	14102	-	138	hypothetical protein
14358	14236	-	123	hypothetical protein
14786	14355	-	432	hypothetical protein
14991	14800	-	192	hypothetical protein
15290	14988	-	303	FIG00629011: hypothetical protein
15493	15302	-	192	hypothetical protein
15784	16095	+	312	Helix-turn-helix motif
16105	16515	+	411	hypothetical protein
16604	17194	+	591	hypothetical protein
17291	18436	+	1146	Phage integrase
18772	19062	+	291	hypothetical protein
20028	19108	-	921	N-acetylmuramoyl-L-alanine amidase (EC 3.5.1.28)
20398	20063	-	336	hypothetical protein
20710	20399	-	312	hypothetical protein
21074	20715	-	360	hypothetical protein
23120	21108	-	2013	BppU family phage baseplate upper protein
23375	23133	-	243	hypothetical protein
26129	23388	-	2742	peptidoglycanDD-metalloendopeptidasefamilyprotein
26830	26126	-	705	hypothetical protein
29208	26836	-	2373	Phage tail length tape-measure protein T
29865	29401	-	465	hypothetical protein
30506	29865	-	642	Phage tail protein
30868	30503	-	366	hypothetical protein
31193	30858	-	336	Phage protein
31503	31183	-	321	minor structural protein, putative
31735	31490	-	246	Phage protein
33067	31898	-	1170	Phage major capsid protein
33738	33064	-	675	hypothetical protein
34900	33719	-	1182	Ammonium transporter
36606	34915	-	1692	Phage terminase, large subunit
36970	36608	-	363	hypothetical protein
37025	36960	-	66	hypothetical protein

Supplementary Table S1;

Table of gene list possessed by *phiEG37k* based on RAST analysis of whole genome sequence**.**

Contig	Position	G0	G2	Gene	Types
NODE_1_length_636754_cov_93.588013	139369	C	T	bifunctional folylpolyglutamate synthase/dihydrofolate synthase	nonsynonymous
NODE_1_length_636754_cov_93.588013	143727	T	C	(non-coding)	-
NODE_1_length_636754_cov_93.588013	157050	G	A	glycoside hydrolase family 125 protein	nonsynonymous
NODE_1_length_636754_cov_93.588013	251380	C	T	hypothetical protein	nonsynonymous
NODE_1_length_636754_cov_93.588013	255002	G	T	MATE family efflux transporter	nonsynonymous
NODE_1_length_636754_cov_93.588013	261677	G	A	hypothetical protein	nonsynonymous
NODE_1_length_636754_cov_93.588013	324729	G	A	hypothetical protein	nonsynonymous
NODE_1_length_636754_cov_93.588013	387729	C	T	M42 family metallopeptidase	nonsynonymous
NODE_1_length_636754_cov_93.588013	513149	G	A	ABC transporter permease	nonsynonymous
NODE_1_length_636754_cov_93.588013	569823	C	T	citrate/2-methylcitrate synthase	nonsynonymous
NODE_2_length_604314_cov_72.335293	19395	T	C	FtsW/RodA/SpoVE family cell cycle protein	nonsynonymous
NODE_2_length_604314_cov_72.335293	21696	T	A	endonuclease MutS2	nonsynonymous
NODE_2_length_604314_cov_72.335293	90416	T	G	(non-coding)	-
NODE_2_length_604314_cov_72.335293	130803	T	G	purine/pyrimidine permease	nonsynonymous
NODE_2_length_604314_cov_72.335293	258997	G	T	(non-coding)	-
NODE_2_length_604314_cov_72.335293	261633	T	A	(non-coding)	-
NODE_2_length_604314_cov_72.335293	263680	T	C	(non-coding)	-
NODE_2_length_604314_cov_72.335293	267202	C	A	(non-coding)	-
NODE_2_length_604314_cov_72.335293	285643	G	A	primosomal protein DnaI	synonymous
NODE_2_length_604314_cov_72.335293	349817	G	A	YvcK family protein	synonymous
NODE_2_length_604314_cov_72.335293	480519	A	G	hypothetical protein	synonymous
NODE_2_length_604314_cov_72.335293	561385	C	T	uracil-DNA glycosylase	nonsynonymous
NODE_2_length_604314_cov_72.335293	592395	C	G	phytoene/squalene synthase family protein	nonsynonymous
NODE_3_length_528442_cov_76.837329	5022	A	C	cation transporter	nonsynonymous
NODE_3_length_528442_cov_76.837329	33747	A	G	(non-coding)	-
NODE_3_length_528442_cov_76.837329	65695	G	T	YitT family protein	nonsynonymous
NODE_3_length_528442_cov_76.837329	118865	C	T	PTS sugar transporter subunit IIC	nonsynonymous
NODE_3_length_528442_cov_76.837329	350715	C	T	starvation-sensing protein RspA	nonsynonymous
NODE_3_length_528442_cov_76.837329	353771	A	C	(non-coding)	-
NODE_3_length_528442_cov_76.837329	418413	A	G	unknown	-
NODE_3_length_528442_cov_76.837329	419401	A	G	unknown	-
NODE_4_length_346645_cov_92.251467	45194	T	C	(non-coding)	-
NODE_4_length_346645_cov_92.251467	51972	C	A	(non-coding)	-
NODE_4_length_346645_cov_92.251467	107254	G	A	LPXTG cell wall anchor domain-containing protein	nonsynonymous
NODE_4_length_346645_cov_92.251467	340503	C	A	hypothetical protein	nonsynonymous
NODE_5_length_233178_cov_105.519644	60309	C	T	DNA-directed RNA polymerase subunit beta'	nonsynonymous
NODE_5_length_233178_cov_105.519644	207799	T	C	transcription antiterminator	synonymous
NODE_6_length_227715_cov_102.686396	120986	G	A	hypothetical protein	synonymous
NODE_6_length_227715_cov_102.686396	185067	G	T	PadR family transcriptional regulator	nonsynonymous
NODE_7_length_208285_cov_77.626615	26118	G	A	helix-turn-helix domain-containing protein	nonsynonymous
NODE_7_length_208285_cov_77.626615	31378	T	A	ABC transporter ATP-binding protein	nonsynonymous
NODE_7_length_208285_cov_77.626615	59176	A	G	(non-coding)	-
NODE_7_length_208285_cov_77.626615	110729	T	C	(non-coding)	-
NODE_7_length_208285_cov_77.626615	117884	C	T	alpha-galactosidase	nonsynonymous
NODE_7_length_208285_cov_77.626615	180623	T	C	MoxR family ATPase	nonsynonymous
NODE_8_length_190413_cov_106.429422	38618	G	A	(non-coding)	-
NODE_8_length_190413_cov_106.429422	52044	C	T	glutamate--tRNA ligase	synonymous
NODE_8_length_190413_cov_106.429422	73873	G	A	PTS trehalose transporter subunit IIBC	nonsynonymous
NODE_8_length_190413_cov_106.429422	86423	T	G	thiol reductant ABC exporter subunit CydD	nonsynonymous
NODE_8_length_190413_cov_106.429422	130883	G	T	FliA/WhiG family RNA polymerase sigma factor	nonsynonymous
NODE_9_length_98316_cov_84.202369	16662	C	T	asparagine synthase (glutamine-hydrolyzing)	nonsynonymous
NODE_9_length_98316_cov_84.202369	24012	T	G	threonine/serine exporter family protein	synonymous
NODE_10_length_93190_cov_97.429119	65403	T	C	(non-coding)	-
NODE_12_length_54283_cov_85.068931	8698	T	G	helix-turn-helix domain-containing protein	nonsynonymous
NODE_12_length_54283_cov_85.068931	46446	A	G	PRD domain-containing protein	nonsynonymous
NODE_14_length_42167_cov_34.028662	53	C	G	phosphopentomutase	nonsynonymous
NODE_15_length_34545_cov_18.546651	26636	A	G	unknown	-
NODE_15_length_34545_cov_18.546651	30780	T	C	unknown	-
NODE_16_length_25828_cov_92.905366	12821	T	C	unknown	-
NODE_20_length_13824_cov_49.410778	9433	C	A	unknown	-

Supplementary Table S2; List of SNPs on core-genome between *Enterococcus gallinarum* strains G0 and G2.

Feature ID	G0 - 1	G0 - 2	G0 - 3	G2 - 4	G2 - 5	G2 - 6	tagwise dispersions - FDR p-value correction*
0610005C13Rik	208.33	225.50	241.00	320.50	308.50	377.33	3.6653E-03
Apoa1	412.50	387.83	104.67	27.67	35.33	47.00	1.5264E-04
Arntl	232.33	163.33	181.00	141.17	108.17	118.00	3.5388E-02
Baiap2l2	558.33	587.50	578.67	721.50	727.33	728.00	2.2159E-02
Bmp2	674.83	646.50	636.17	791.67	828.50	891.50	2.9529E-02
C3	2429.83	3857.00	2560.50	1455.50	2067.17	1433.67	3.6653E-03
Cldn8	333.00	284.83	271.83	184.50	181.50	175.67	3.8762E-06
Defa24	38.83	0.00	8.50	0.00	0.00	0.00	1.6368E-02
Eln	257.67	230.00	240.33	180.83	158.33	136.83	1.6583E-03
G6pc	7.50	17.33	7.17	1.67	0.50	0.00	1.0710E-02
Gm21857	44.17	9.17	35.50	6.17	7.00	6.17	2.6955E-02
Igfbp6	206.17	212.50	259.00	143.67	166.33	135.83	1.4491E-02
Ighv1-66	1.00	13.67	66.50	1.00	0.00	1.17	1.6432E-02
Ighv5-12	1.00	0.00	1.50	0.00	0.00	65.50	4.9774E-02
Ighv5-17	5.17	2.67	1.50	168.83	7.17	4.00	2.0937E-02
Igkv1-133	0.00	1.00	0.00	1.00	0.00	87.50	4.1259E-03
Igkv12-46	171.00	1.67	26.33	2.17	1.67	7.50	2.4564E-02
Igkv4-57-1	8.50	26.33	11.17	1.00	1.67	4.00	1.4491E-02
Lrp2	24.17	15.67	18.50	8.50	6.17	4.00	2.4644E-02
Lrrn4	31.17	47.00	49.00	21.33	23.33	10.17	3.9268E-02
Msln	68.17	64.33	94.33	44.50	33.67	22.50	2.6428E-03
Muc2	125961.83	105549.17	105549.17	125961.83	125961.83	125961.83	1.4491E-02
Muc3	11911.33	9730.00	7668.17	14702.67	13385.50	15812.33	2.3076E-02
Muc3a	527.83	471.17	423.33	797.83	626.50	811.33	6.3473E-04
Muc4	1473.00	1382.17	1305.33	1876.33	1668.17	1788.67	2.9900E-02
Myrf	40.00	41.00	47.83	15.50	21.17	16.83	1.6583E-03
Npas2	302.17	227.83	196.00	168.83	143.50	116.00	2.2159E-02
Per2	318.67	384.00	395.67	682.17	618.00	637.33	1.2217E-10
Per3	175.83	224.50	196.00	336.00	334.67	374.50	1.4625E-07
Scd2	4242.17	5520.50	4506.00	5957.17	6348.50	6871.17	4.1259E-03
Serpine1	53.33	82.50	56.33	18.50	21.33	34.33	6.3473E-04
Tef	1131.83	1549.83	1462.17	1973.50	2093.50	2020.00	4.8374E-04
Tsku	484.17	500.33	466.33	578.67	705.83	688.17	1.5845E-02
Upk3b	80.17	88.67	102.83	48.67	38.17	42.67	3.2874E-05

Supplementary Table S3;
A List of normalized expression level of genes which were significantly changed in *phiEG37k* – colonized colon (G0) compared to uncolonized colons (G2). * FDR p-values were calculated by EDGE.

Antigen	Clone	Fluorescence	Company
I-A^b	AF6-120.1	Alexa Fluor 488	BioLegend
CD11c	N418	Brilliant violet 421	BioLegend
CD103	2E7	biotin-labled	BioLegend
Streptavidin	-	PE-CF594	BD Bioscience
CX3CR1	SA011F11	PE-Cy7	BioLegend
CD80	16-10A1	PE	BioLegend
CD3	17A2	Pacific blue	BioLegend
CD4	GK1.5	PECy7	BioLegend
CD8	53-5.8	PE	BioLegend
IL-17A	TC11-18H10.1	PerCP/Cyanine5.5	BioLegend
IFNg	R4-6A2	FITC	BioLegend
Foxp3	FJK-16s	APC	eBioscience

Supplementary Table S4; List of antibodies used for flow cytometric analysis

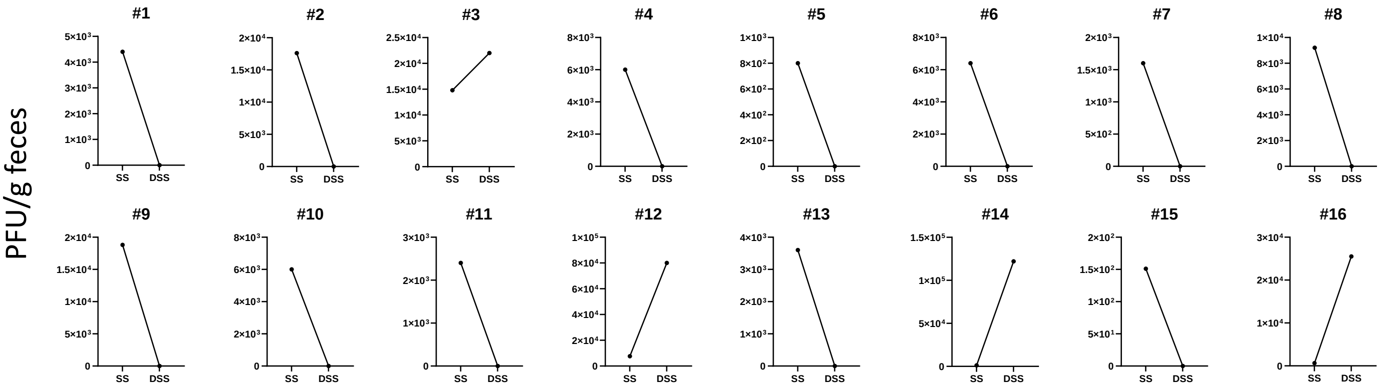

Supplementary Figure S1; Alteration in the abundance of fecal VLPs by DSS colitis.

The PFU of VLPs related with 16 *Enterococcal* bacterial clones, which showed sensitivity to infection by fecal VLP at the first screening plaque assay. The PFU of individual clones is displayed in separate graphs. All the line graphs were combined in one graph in Fig. 1b. *phiEG37k* was isolated by plaque assay with *Enterococcal* bacterial strain #16 (*G0*).

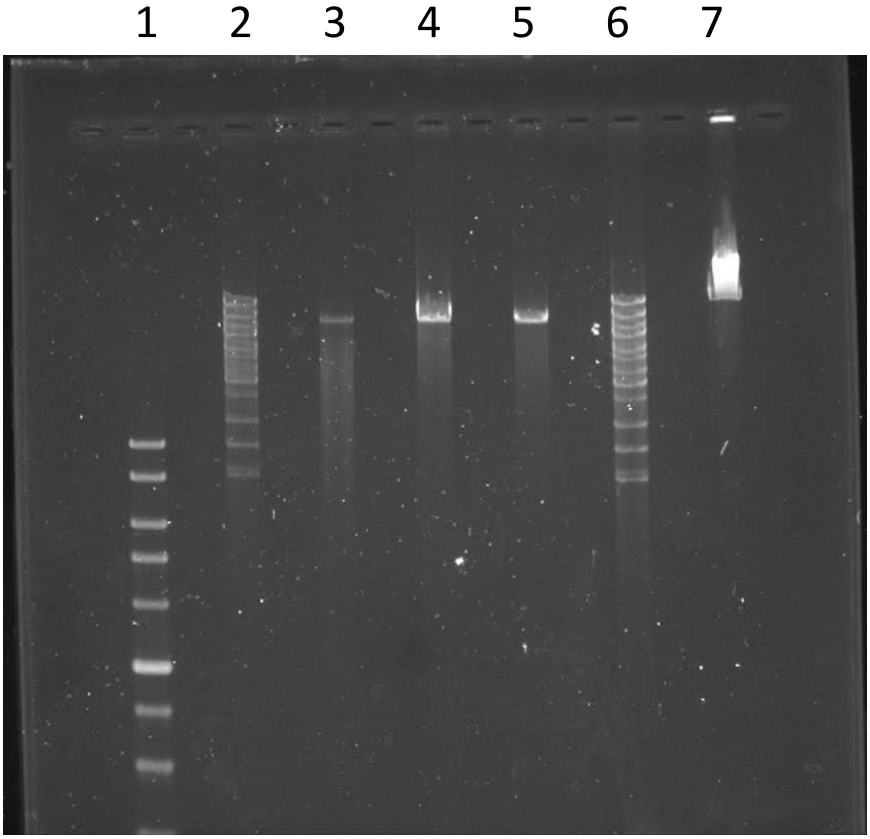

Supplementary Figure S2 ; Full-length gel image of Figure 1e.
Lane 1;1kb ladder, Lane2 and Lane 6 ;High Molecular Weight DNA Marker (Invitrogen), Lane 3-5;*phiEG37k* and Lane 7;λDNA.

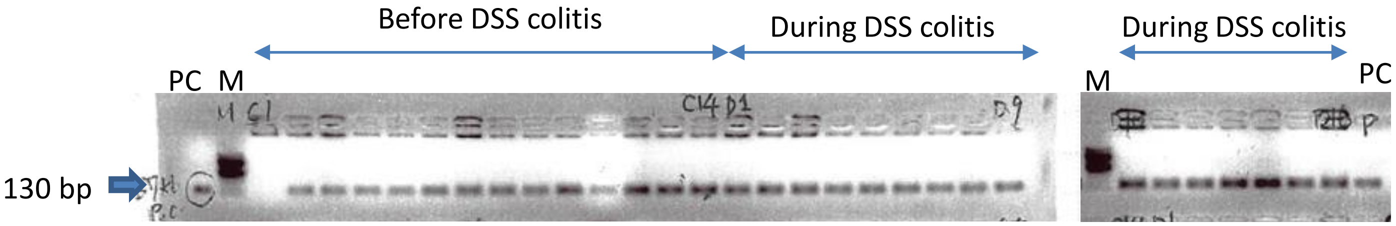

Supplementary Figure S3 ; PCR analyses of of genomes of different clones of *E. gallinarum* by *phiEG37k*-specific primer. The genomes are extracted from feces of C57BL/6 mice before or during DSS-induced colitis. Bands of 130 base pair (bp) size of PCR products, which are the same size as that of PCR products generated from purified phiEG37k (PC), were detected in the lanes of the individual positive samples. The images of the agarose gels were cropped from the image of the same gel and show almost full-length gels. PC; PCR product which was generated by amplifying the genome of purified *phiEG37k* with *phiEG37k*-specific primer (positive control), M; Molecular weight marker, 100bp DNA ladder

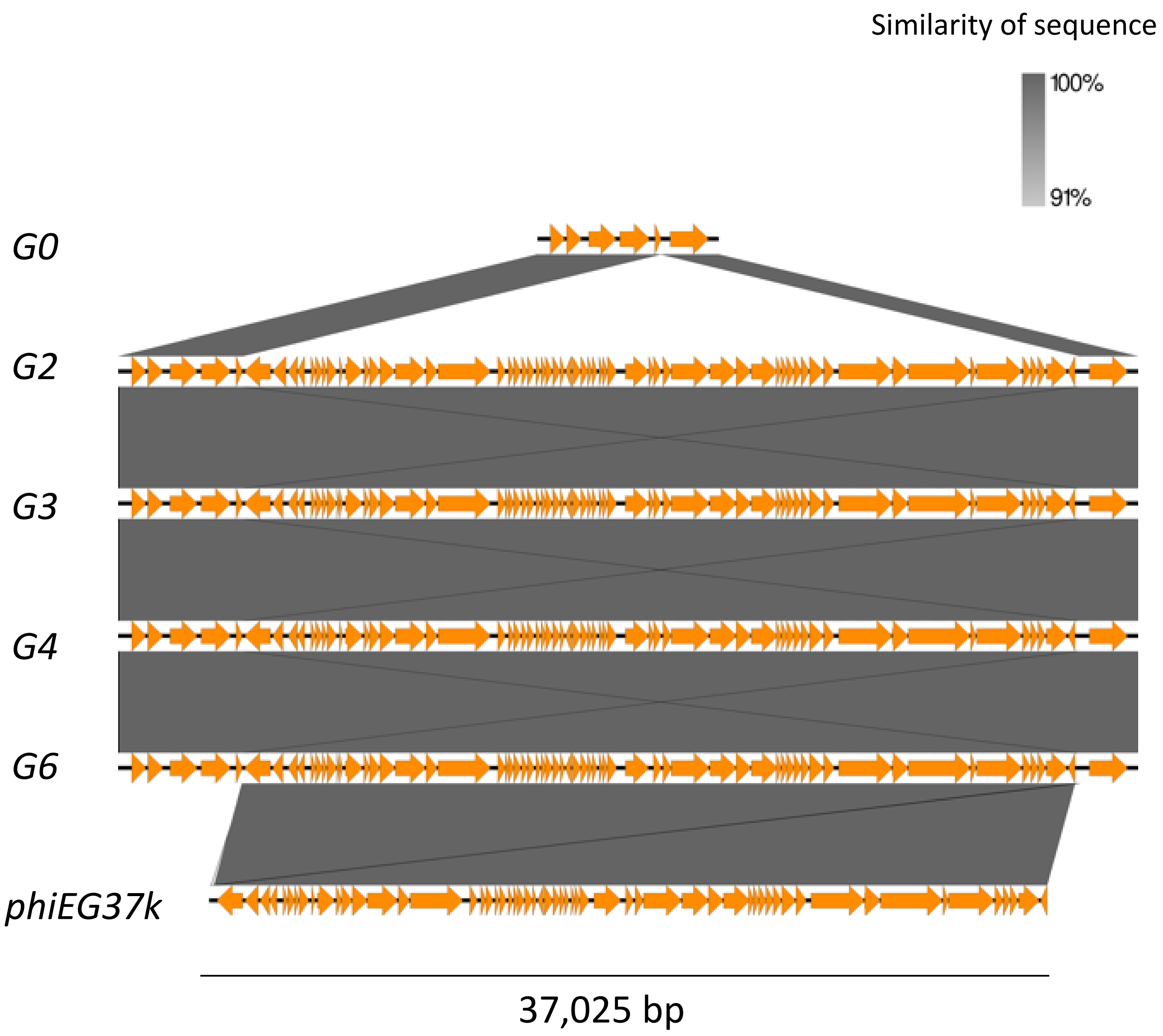

Supplementary Figure S4; **Analysis of *phiEG37k* integration sites of the four clones of gut *E. gallinarum* by draft whole-genome sequencing**

Displayed is only around the phage genome insertion site. Orange arrows indicate CDSs shown in Supplementary Table S1. The gray-color intensity indicates sequence similarity. All the E. gallinarum strains, G2, G3, G4 and G6, integrates phiEG37k genome at the same site with highly conserved sequence.

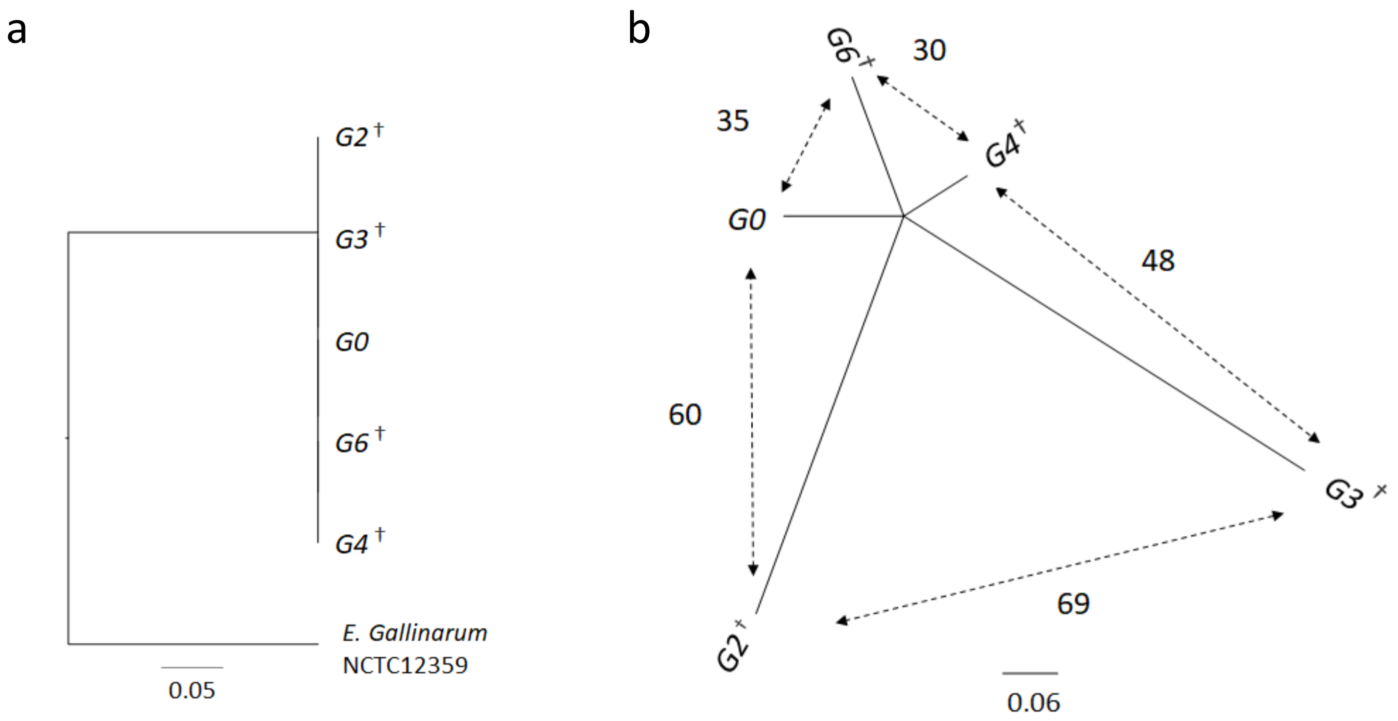

c

	G0	G2	G3	G4	G6
G0	0	60	53	25	35
G2	60	0	69	55	65
G3	53	69	0	48	58
G4	25	55	48	0	30
G6	35	65	58	30	0

Supplementary Figure S5 ; Phylogenetic tree based on core genome and Single Nucleotide Polymorphisms (SNPs) among *E. gallinarum*.

(a)Phylogenetic tree created by analyzing the draft whole genome data of a type strain *E. gallinarum* NCTC12359 (accession no. GCA_900447935.1) to *E. gallinarum* G0, G2, G3, G4, and G6.

(b) Phylogenetic tree and SNPs of *E. gallinarum* G0, G2, G3, G4, and G6 only. The presence or absence of phiEG37k does not affect the results of phylogenetic analysis because *phiEG37k* is not included in the core genome. The length of bars indicates an amount geneic change of each number. (c) Number of SNPs between individual *E. gallinarum* strains. †,lysogenic strain bearing *phiEG37k*

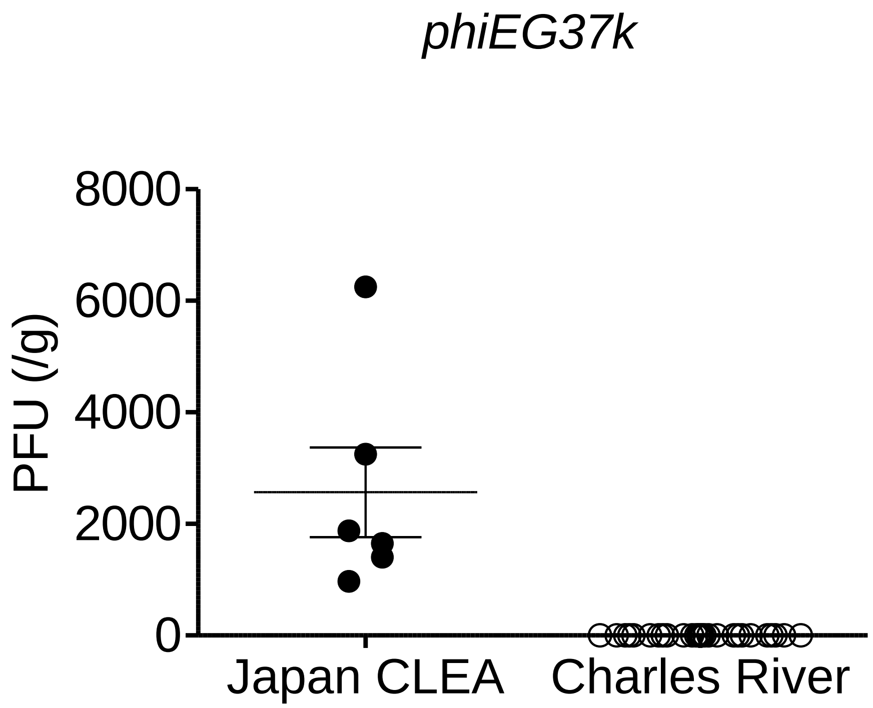

Supplementary Figure S6; Fecal titer of *phiEG37k* phage from C57BL/6 mice from two different vendors (Clea, n=6; Charles River Laboratories Japan, n=29)**.** Fecal titer was measured within a week after the mice had shipped from the individual vendors to our facility.

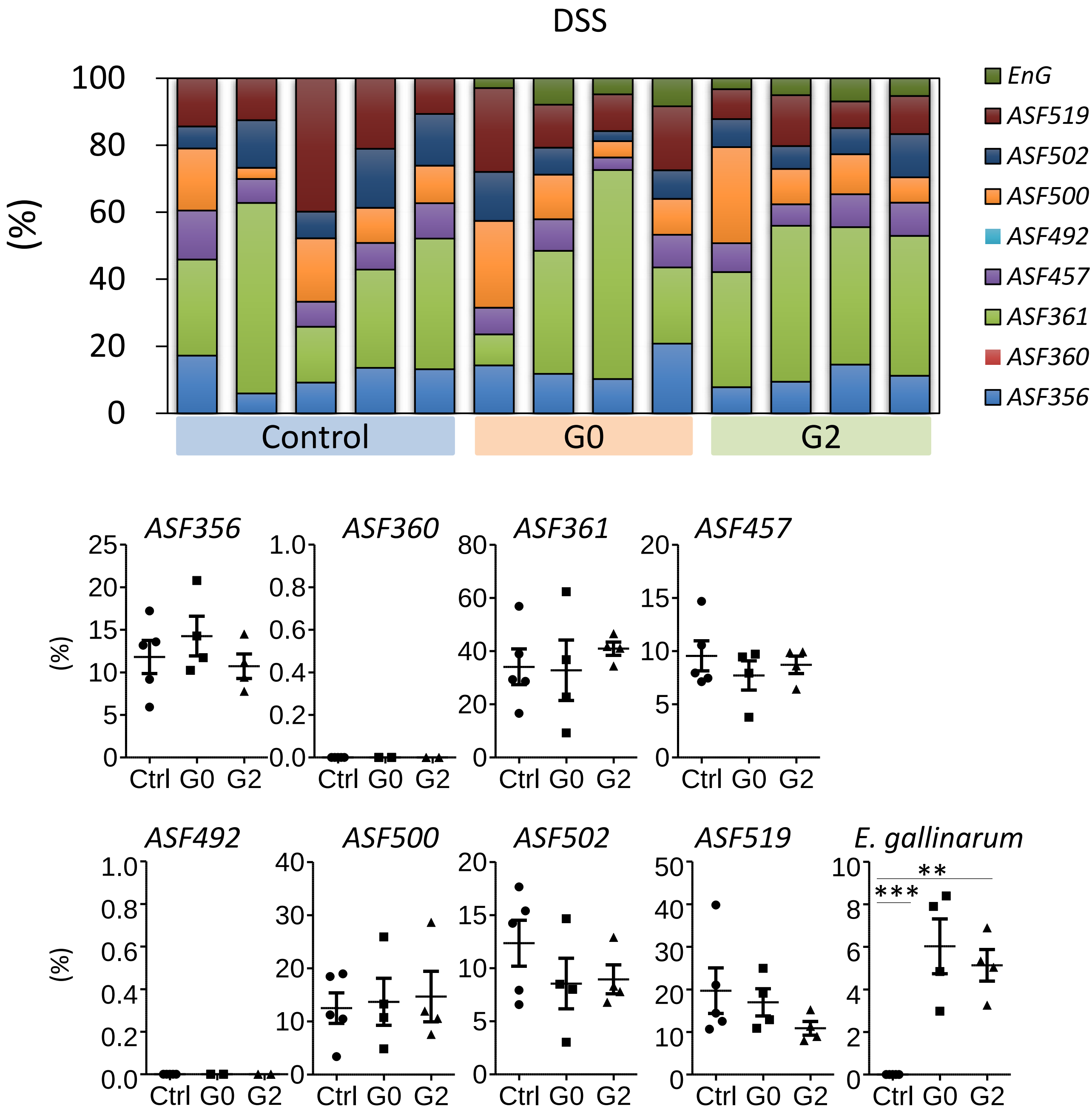

Supplementary Figure S7; Components of colonic microbiota in ASF-mice without gavage or with gavage of G0 or G2 (G0 or G2, respectively).
EnG; *E. gallinarum*, **P < 0.01, ***P < 0.001

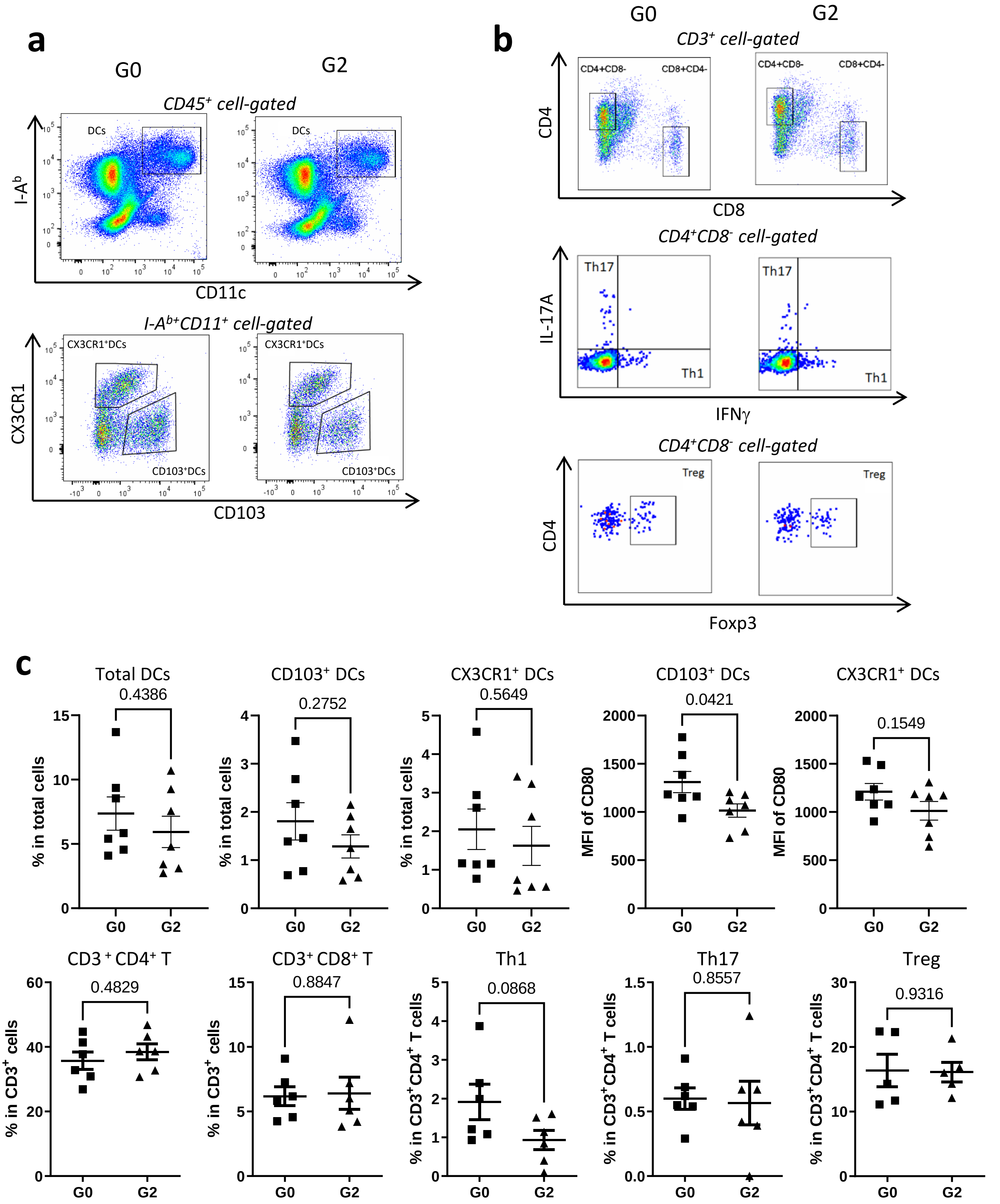

Supplementary Figure S8; Flow-cytometric analysis of colonic lamina propria cells.

Colonic lamina propria cells were isolated from G0- or G2-colonized ASF mice and subjected to flow-cytometric analysis for lymphocytes and dendritic cells (DCs). (a), (b) Representative cytograms of dendritic cells (a) and T lymphocytes (b). (c) Pooled data of the frequency of individual populations and mean fluorescence intensity (MFI) of CD80 of DCs. Student's t-test was performed. Error bars show standard errors.

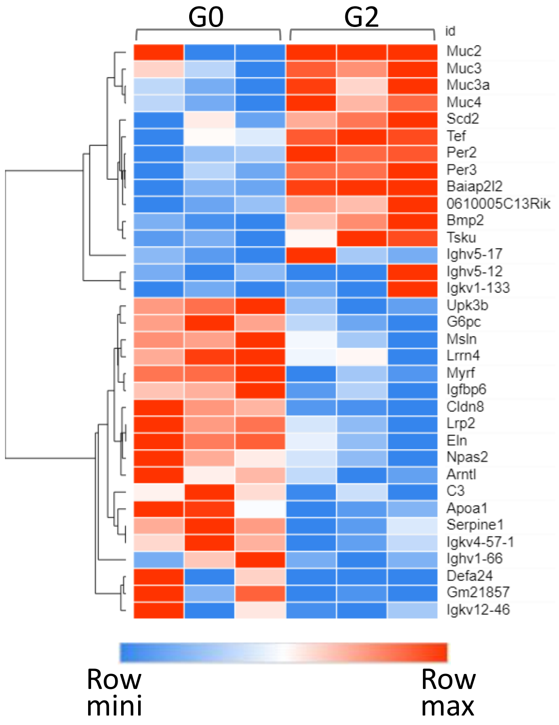

Supplementary Figure S9; RNA-seq analysis of colonic tissue from G0- or G2-colonized ASF-mice in steady state.

Differentially expressed genes with p-value less than 0.05 were listed up. A relative color scheme uses the minimum and maximum values in each row to convert values to colors. n=3 each.

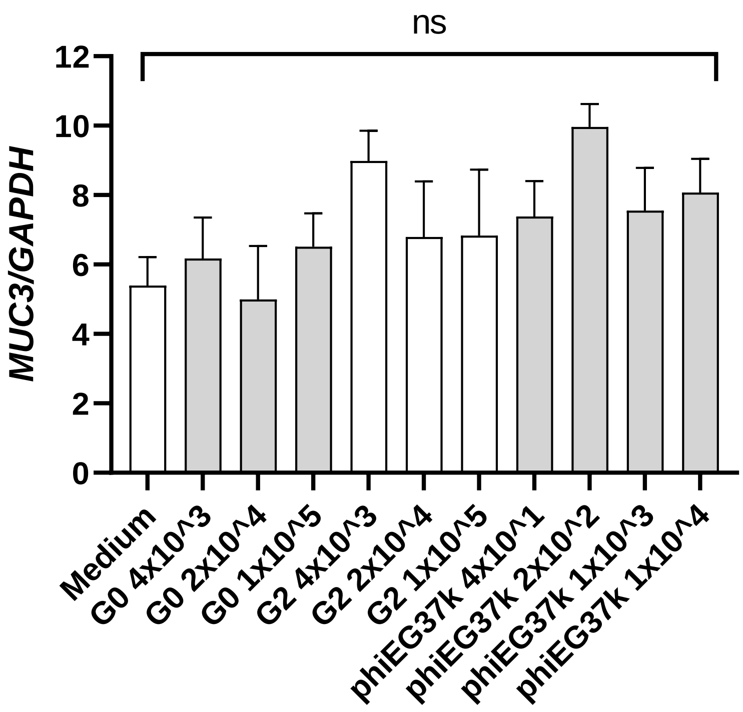

Supplementary Figure S10; *MUC3* gene expression of Caco-2 cells co-cultured with *G0*, *G2* or *phiEG37k*.

90 % confluent Caco2 cells were plated in 24-well plate and co-incubated with *G0*, *G2* or *phiEG37k* with indicating numbers of cells or viral particles for 4 hours. Total RNA was extracted and qRT-PCR of *MUC3* was performed. The error bars indicate standard error.

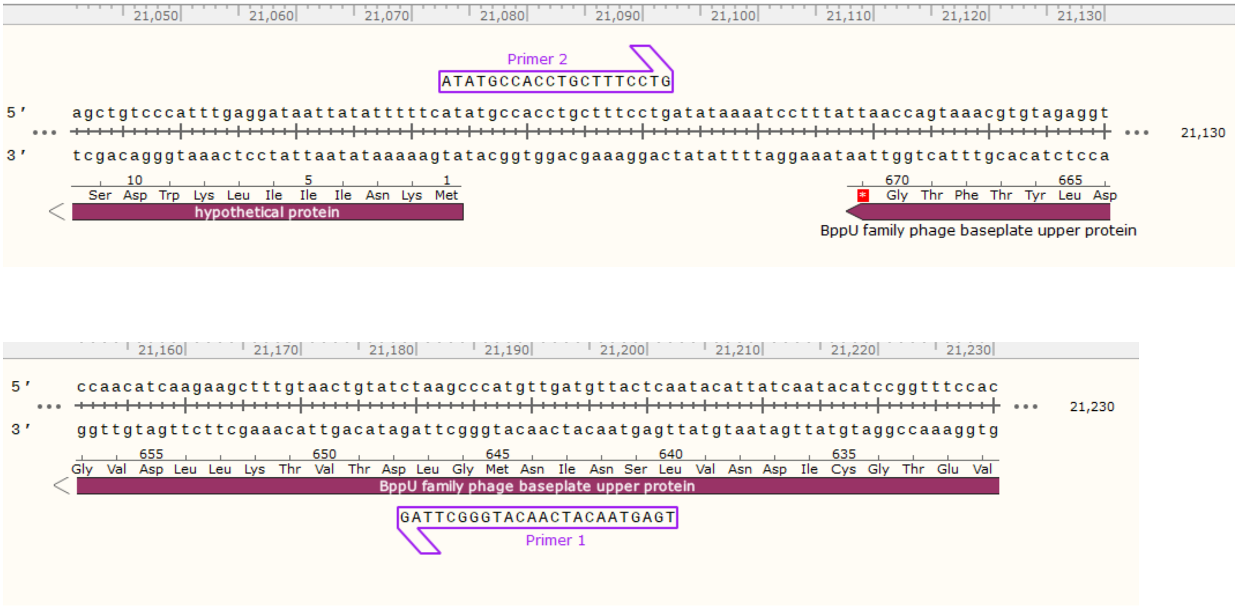

Supplementary Figure S11; Positions of PCR primer for *phiEG37k*.
